# The Tumor Suppressor Gene TUSC2 (FUS1) Sensitizes NSCLC to the AKT Inhibitor MK2206 in LKB1-dependent Manner

**DOI:** 10.1371/journal.pone.0077067

**Published:** 2013-10-17

**Authors:** Jieru Meng, Mourad Majidi, Bingliang Fang, Lin Ji, B. Nebiyou Bekele, John D. Minna, Jack A. Roth

**Affiliations:** 1 Section of Thoracic Molecular Oncology, Department of Thoracic and Cardiovascular Surgery, The University of Texas MD Anderson Cancer Center, Houston, Texas, United States of America; 2 Department of Biostatistics, The University of Texas MD Anderson Cancer Center, Houston, Texas, United States of America; 3 Hamon Center for Therapeutic Oncology Research and Simmons Cancer Center, The University of Texas Southwestern Medical Center at Dallas, Dallas, Texas, United States of America; University Magna Graecia, Italy

## Abstract

TUSC2-defective gene expression is detected in the majority of lung cancers and is associated with worse overall survival. We analyzed the effects of TUSC2 re-expression on tumor cell sensitivity to the AKT inhibitor, MK2206, and explored their mutual signaling connections, *in vitro* and *in vivo*. TUSC2 transient expression in three LKB1-defective non-small cell lung cancer (NSCLC) cell lines combined with MK2206 treatment resulted in increased repression of cell viability and colony formation, and increased apoptotic activity. In contrast, TUSC2 did not affect the response to MK2206 treatment for two LKB1-wild type NSCLC cell lines. In vivo, TUSC2 systemic delivery, by nanoparticle gene transfer, combined with MK2206 treatment markedly inhibited growth of tumors in a human LKB1-defective H322 lung cancer xenograft mouse model. Biochemical analysis showed that TUSC2 transient expression in LKB1-defective NSCLC cells significantly stimulated AMP-activated protein kinase (AMPK) phosphorylation and enzymatic activity. More importantly, AMPK gene knockdown abrogated TUSC2-MK2206 cooperation, as evidenced by reduced sensitivity to the combined treatment. Together, TUSC2 re-expression and MK2206 treatment was more effective in inhibiting the phosphorylation and kinase activities of AKT and mTOR proteins than either single agent alone. In conclusion, these findings support the hypothesis that TUSC2 expression status is a biological variable that potentiates MK2206 sensitivity in LKB1-defective NSCLC cells, and identifies the AMPK/AKT/mTOR signaling axis as an important regulator of this activity.

## Introduction

The complete loss or reduction of expression of the tumor suppressor gene TUSC2 (tumor suppressor candidate 2), also known as FUS1, is detected in 82% of non-small cell lung cancers (NSCLC) and 100% of small cell lung cancers (SCLC) [1]–[3]. TUSC2 gene product, a multifunctional protein, plays an important role in various cellular processes, including transcription, cell-cycle progression and apoptosis [4]. Recently, we have demonstrated that exogenous expression of TUSC2 in non-small cell lung carcinoma cells, deficient of its expression, significantly inhibited tumor cell growth [5], [6]. In addition, intravenous systemic delivery of TUSC2 to distant tumors, via intravenous N-[1-(2,3-dioleoyloxy)propyl]-N,N,N-trimethylammonium chloride (DOTAP): cholesterol nanovesicles, suppressed tumor growth and progression in orthotopic human lung cancer xenograft models, and in a phase I clinical trial of stage 4 lung cancer patients who had progressed on chemotherapy [4]–[7].

Results from our previous studies have showed that TUSC2 inhibited functions of protein tyrosine kinases including EGFR, PDGFR, AKT, c-Abl, and c-Kit. TUSC2 activated the apoptotic protease activating factor 1 (Apaf-1), a transcriptional target of p53 and a key factor in the mitochondrial apoptotic pathway [8]–[12]. In addition, co-expression of TUSC2 and p53 profoundly suppressed tumor cell growth and caused apoptosis in both p53-sensitive and p53-resistant NSCLC cell lines [8].

A variety of small molecule drugs have been used to target specific signaling pathways in lung cancer [13]–[15]. Contrary to normal cells, tyrosine kinase (TK) activity in tumor cells is disrupted in such a way that constitutive signaling triggers tumorigenesis. TK inhibitors (TKIs), particularly proteins that target the epidermal growth factor receptor pathway (EGFR), have a promising therapeutic value for the treatment of lung cancer [16], [17]. Unfortunately, TKI–resistant disease is often acquired, leading to tumor progression [18]–[20]. This problem stems from the complex interconnection between deregulated receptor tyrosine kinase (RTKs) pathways with similar targets. Therefore, targeting common pathways, rather than multiple receptors, may be a more effective strategy for preventing or overcoming drug resistance.

The AKT/mTOR intracellular pathway is an important regulator of apoptosis [21]. This pathway is a relevant therapeutic target in lung cancer because it serves as a convergence for many growth stimuli. Deregulated activation of AKT has been associated with several tumorigenic properties and occurs in 70% of NSCLC. These include cellular transformation, promotion of tumor invasion and angiogenesis, and resistance to chemotherapy and radiation therapy [22]–[24]. Among the major signaling targets of Akt, is activation of the mammalian target of rapamycin (mTOR), which regulates protein synthesis, cell proliferation, and other cellular functions [25]. One of the kinases that mediate mTOR activation by Akt is the AMP-activated protein kinase (AMPK). Under depleted energy conditions, this pleotropic protein is vital in keeping cell metabolism and growth at normal levels [26]. AMPK regulates apoptosis and cell cycle progression through p53, and is activated by the tumor suppressor LKB1 [26], [27]. The latter is inactivated or mutated in up to 50% of NSCLC.

We have already showed the role of AKT upregulation in mediating resistance of NSCLC to the inhibitor of the MEK1/2 kinases, AZD6244 [24]. In this study, we investigated whether restoration of TUSC2 gene expression could increase sensitization to the specific AKT inhibitor, MK2206, *in vivo* and *in vitro*. We provide several lines of biological and biochemical evidence for cooperation between TUSC2 and MK2206 in suppressing growth of LKB1-defecive NSCLC cells.

## Materials and Methods

### Reagents

MK2206, synthesized by Dr. William G. Bornmann (M.D. Anderson Cancer Center, Houston, TX), was dissolved in DMSO to concentrations of 20 mM and stored at −80°C. AMPK, AKT, mTOR, p-AMPK (Th172), p-AKT (Ser473), p-AKT (Th308) and p-mTOR (Ser2448) antibodies were purchased from Cell Signaling Technology (Danvers, MA). Rabbit anti-TUSC2 poylclonal antibody was developed against a synthetic oligopeptide (gasgskarglwpfasaa) derived from the N-terminal amino-acid sequence of the TUSC2 protein (Bethyl Laboratories, Montgomery, TX). Protease inhibitor cocktail, β-actin antibody, and sulforhodamine B were obtained from Sigma Chemical Corporation (St. Louis, MO). Protein assay materials were purchased from Bio-Rad Laboratories (Hercules, CA). Lipofactamin ™ 2000 was purchased from Invitrogen Corporation (Carlsbad, CA). DOTAP and cholesterol were purchased from Avanti Polar Lipids (Albaster, AL). Synthesis and preparation of DOTAP-Cholesterol (DC) were described previously [6]. AMPK siRNA was purchased from Santa Cruz Biotechnology (Santa Cruz, CA).

### Transient transfection and RNA interference induction

Cell lines used in this study were obtained from the Hamon Center for Therapeutic Oncology Research at UT Southwestern Medical Center. HCC366 was established by the Hamon Center for Therapeutic Oncology Research at UT Southwestern Medical Center. A549, H322, H358 and H661 were established at the National Cancer Institute. Cells were maintained in RPMI 1640, supplemented with 10% fetal bovine serum, 100 µg/mL ampicillin, and 0.1 mg/mL streptomycin, and cultured at 37°C in a humidified atmosphere containing 5% CO_2_ and 95% air. Cells were plated in a six-well plate at 2×10^5^ cells/well, and TUSC2 transfection was carried out with 2 µg DOTAP:cholesterol (DC) –TUSC2 encapsulated plasmid DNA nanovesicles in serum-free medium, as previously described [6]. Cells transfected with empty vector served as controls. Transfection efficiency was assessed with a green fluorescent protein (GFP)–expressing plasmid vector. AMPK gene knockdown was accomplished by transfecting cells with 50 nM AMPK-specific siRNA with Lipofectamine™ 2000 according to the manufacturer's protocol. The siRNA and Lipofectamine were diluted with Opti-MEMI medium (Invitrogen), combined together, and incubated for 20 min before adding the mixture to the cells. To determine whether siRNA blocked AMPK expression, cells were harvested 48 h posttransfection for evaluation of AMPK proteins by Western blotting.

### Cell Viability Assay

TUSC2, and/or MK2206 antitumor activity on tumor cell viability was analyzed by SRB assay-based cell count (Sigma). Briefly, cells were plated in 96-well microliter plates at a density of 3,000 cells per well and transfected with TUSC2 plasmid-based nanovesicles. Twenty-four hours post-transfection, cells were starved for 24 hours before treatment with MK2206 at the indicated concentrations. Cell viability was quantified after 48 hours in a microplate reader using the sulforhodamine B (SRB) assay according to the manufacturer's instructions. The percentage of viable cells was calculated using the equation OD_T_/OD_C×_×100%, where OD_T_ and OD_C_ represented the absorbance of the treatment group and the absorbance of the control group, respectively. The median inhibitory concentration (IC_50_) values were determined using CurveExpert 1.3 software and plotted in dose-response curves.

### Colony Formation Assay

TUSC2 and MK2206 combined effects on tumor cell colony formation were assessed as follows: Cells were seeded at 5×10^5^ cells/per 60 mm^2^ plate one day prior to transfection with TUSC2-expressing and control plasmid vector DNAs. Two micrograms of expression vector were co-transfected with 1 µg of the neomycin-resistant gene-containing pcDNA3 vector. Twenty four hours post-transfection, cells were split, replated in triplicate, and grown in medium containing 400 µg/ml of the antibiotic G418 before treatment with 1 μ MK2206. Only a fraction of seeded cells retained the capacity to produce colonies. Next, surviving colonies were fixed with glutaraldehyde (6.0% v/v), stained with crystal violet (0.5% w/v), and counted using a stereomicroscope. Colony-forming cells were determined using the Image-J software.

### Cell Apoptosis Assay

Cell apoptotic activity was measured by flow cytometry using propidium iodide containing 30 µg/ml RNase. In brief, at the indicated time, after 2 µg TUSC2 plasmid transfection and/or 1 µM MK2206 treatment, cells were fixed in 4% paraformaldehyde, permeabilized with 70% ethanol, washed with PBS, and stained with propidium iodide solution containing 40 ug/mL propidium iodide and 10 ug/mL DNase-free RNase A. DNA fragmentation was analyzed by flow cytometry, and relative apoptotic cells were calculated in terms of the FITC-positive values in cells.

### Caspase Assay

Caspase-9 activity was assessed by cleavage of caspase-9-specific substrates using western blot. Cells were lysed in laemmli sample buffer (Bio-Rad Laboratories, Hercules, CA), and 30 µg of total proteins, from each lysate, were separated by electrophoresis in 10% SDS PAGE, transferred to polyvinylidene fluoride membranes (Millipore, Marlboro, MA), and probed with caspase-9 antibody (Cell Signaling Technology, Danvers, MA). The latter detects both full length caspase-9 (47 kDa) and large fragments of caspase-9 (35 kDa, 37 kDa). Immunoreactive bands were visualized with Odyssey Imager (Li-COR Biosciences, Lincoln, NE).

### *In vitro* kinase assay

The kinase activity of AKT and mTOR were measured with K-LISA AKT and mTOR assay kit (Calbiochem, San Diego, CA) respectively, which are enzyme-linked immunosorbent assays (ELISA). AKT and mTOR proteins were immunoprecipitated using AKT and mTOR specific antibodies. The immune complexes were assayed for AKT and mTOR kinase activities using GSK-3α and p70S6K GST fusion proteins as substrates, respectively. Substrate absorbance and reference wavelengths were measured at 450 nm and 540/595 nm, respectively, using a Synergy 2 Multi-detection microplate reader (BioTek Instruments).

AMPK activity was assessed with SAMS peptide. In brief, AMPK was immunoprecipitated from 200 ug cell lysis with anti-α1 AMPK antibody. Kinase activity in the immunocomplexes was measured by phosphorylation of SAMS peptide with 10 µCi of (γ-^32^P) ATP and 20 µM SAMS peptide. After incubation at 30°C for 10 min, reaction mixtures were spotted on P81 phosphocellulose paper, washed with 0.75% phosphoric acid and acetone, and the radioactivity of phosphorylated SAMS peptide was quantified by scintillation counting.

### Animal Studies

A subcutaneous xenograft mouse model of the human NSCLC H322 cell line was used to evaluate the combined effects of TUSC2 re-expression and MK2206 treatment *in vivo*. All animal experiments were carried out following approval by MD Anderson institutional review board, and were performed in accordance with the Guidelines for the Care and Use of Laboratory Animals published by the National Institutes of Health. The mice used were nu/nu female (6–8 weeks old) purchased from Charles River Laboratories (Wilmington, MA). A total of 3×10^6^ H322 cells were inoculated subcutaneously into the right dorsal flanks of the nude mice. When tumors reached an average volume of about 0.1 cm^3^, mice were randomly divided into control and treatment groups (n = 5 animals per group). The treatment groups were: DC–EV complex; DC–TUSC2 complex; MK2206 alone; and DC–TUSC2 complex plus MK2206. Animals were intravenously injected three times a week for 3 weeks with systemic DC–based nanovesicles at a dose of 25 µg of plasmid DNA and 10 nmol DC in 100 µL of 5% dextrose in water per mouse. MK2206, solubilized in a medium containing 0.5% hydroxypropyl methylcellulose and 0.1% polysorbate buffer, was oral fed at 50 mg/kg, three times a week for 3 weeks. Based on doses previously used in a clinical trial, we choose 50 mg/kg 3 times a week [15]. Tumor size, measured by calipers, was recorded every 3 days. Tumor volumes were calculated by taking length to be the longest diameter across the tumor and width to be the corresponding perpendicular diameter, using the following formula: length × width^2^×0.52. Tumor growth inhibition rate was calculated as 100%× (tumor size_treated_/tumor size_control_) on each measurement day. All mice were weighed three times per week, and daily food and water consumption were monitored. No animal died during the experimental period. Eight hours after the last dose of MK2206 and 21 days following the first treatment, animals were sacrificed by CO_2_ inhalation and tumors were resected, fixed with 4% paraformaldehyde, paraffin-embedded for immunohistochemistry staining with the indicated antibodies. Tissue sections were examined under a Nikon TC200 fluorescence microscope equipped with a digital camera.

### Statistical analysis

All experiments were repeated at least three times in duplicate or triplicate samples. Data from *in vitro* and *in vivo* studies were expressed as the mean ± standard deviation with 95% confidence inter vals. The statistical significance of differences between TUSC2 or MK2206, alone, and TUSC2 plus MK2206 combined treatment was performed by t-test and two-way ANOVA using GraphPad Prism and JMP software. The results are considered significant at *P*<0.05.

## Results

### Inhibition of tumor cell viability and colony formation by forced expression of TUSC2 combined with MK2206 in TUSC2/LKB1-defective NSCLC cell lines

**Figure 1:**
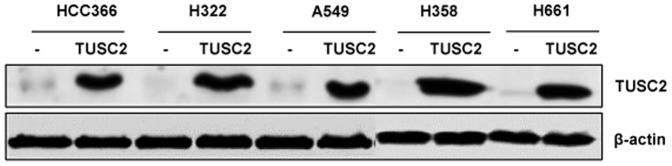
Endogenous and overexpressed levels of TUSC2 in LKB1-defective and wild type NSCLC cells. Cells were transfected with DC-TUSC2 for 24 hours, and TUSC2 protein levels were detected by western blot with a rabbit anti-TUSC2 polyclonal antibody.

**Figure 2:**
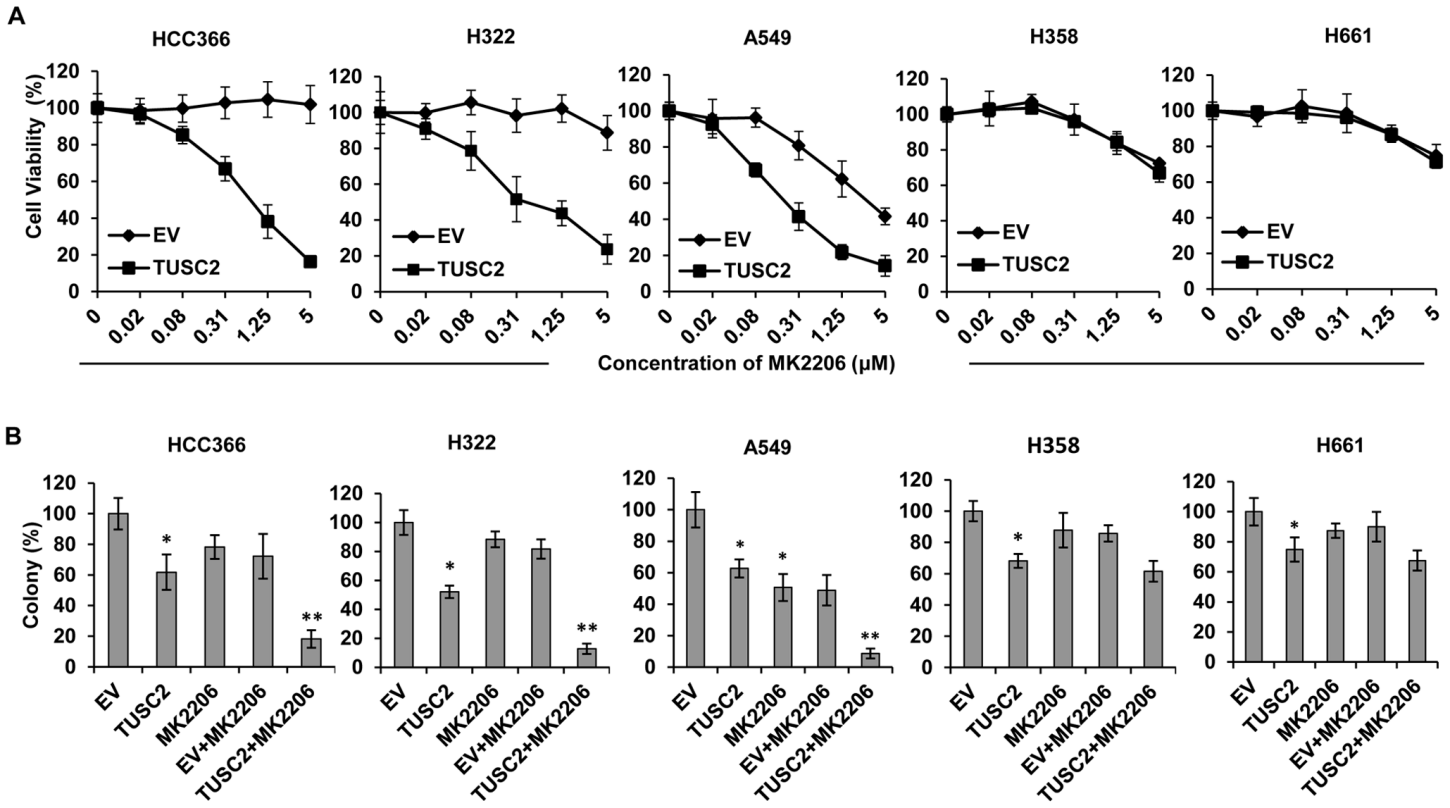
Inhibition of tumor cell viability and colony formation by TUSC2 transfection and MK2206 combined treatment in LKB1-defective and wild type NSCLC cells. A) Forty-eight hours post-treatment, cells were assayed for viability as described in Materials and Methods. Cell viability was plotted against concentration of MK2206. B) Cells were transfected with DC-TUSC2. Twenty four hours post-transfection, cells were split, replated in triplicate, and grown in medium containing 400 µg/ml of the antibiotic G418 before treatment with 1 µM MK2206. Colonies were fixed with glutaraldehyde (6.0% v/v), stained with crystal violet (0.5% w/v), and counted using a stereomicroscope. Columns, mean of three different experiments, each with duplicate samples; bars, SD. *, *P*<0.05, compared with EV control; **, *P*<0.05, compared with EV+MK2206

Five human NSCLC cell lines, with known EGFR, K-ras, B-raf, PI3K, LKB1 and TUSC2 (shown as 3p abnormalities) gene status, were evaluated for sensitivity to MK2206 after TUSC2 transfection (Table 1). The gene mutation status of these cell lines were obtained from online database (http://www.sanger.ac.uk/genetics/CGP/CellLines/). HCC366, H322, and A549 cells are LKB1-deficient, and H358 and H661 are LKB1-wild type. TUSC2 gene expression was assessed by Western Blot analysis (Fig.1). First, viability assays were performed on cells transfected with 2 µg of TUSC2 plasmid alone. TUSC2 induced inhibition of cell growth in all five tested cell lines (data not shown). Next, viabilities were assessed in cells treated with MK2206, alone, for forty-eight hours at concentrations ranging from 0.019 to 5 µM. As Figure 2A shows, at 1 µM of MK2206, viabilities of HCC366, H322, A549, H358, and H661 cells were 100%, 100%, 62%, 84%, and 87%, respectively. However, in LKB1-defecive HCC366, H322, and A549 cells, the combination of TUSC2 transient transfection and MK2206 treatment significantly repressed their growth and survival (*P*<0.05). This effect was observed at nanomolar MK2206 concentrations. At 1 µM MK2206, the relative survival rates of HCC366, H322, A549 cells were reduced by 65%, 60%, and 80%, respectively. Their IC_50_ values were reduced by 16.5, 8.5, and 5.1 fold, respectively (Table 1). Similarly, colony formation by these cells was markedly inhibited. Their ability to form colonies was reduced by 89%, 87%, and 91%, respectively (Fig. 2B). In contrast, survival and colony formation of the LKB1-wild type H358 and H661 cells were not significantly affected by TUSC2-MK2206 combined treatment (Fig. 2A, 2B) suggesting that LKB-1 gene status is associated with sensitivity to the combined treatment.

**Table 1:** IC_50_ values and gene mutation status of five lung cancer cell lines.

Cell Line	Tumor Subtype	KRAS	BRAF	EGFR	PIK3CA	LKB1	3p abnormalities	IC_50_ (MK2206 alone)	IC_50_ (MK2206+TUSC2)
H322	BA	wt	wt	wt	wt	E98-G155 deletion	inv(3p13)	20.39	1.24
HCC366	ADSQ	wt	wt	wt	wt	-2.5 Kb∼exon1 deletion	unknown	18.4	2.17
A549	AD	c,35G>A;p,G12S	wt	wt	wt	109C>T; Q37Ter	unknown	2.86	0.56
H358	BA	(c,35G>T;p,G12C),(c,35>T;p,G12V)	wt	wt	wt	wt	del(p14-p23), inv (3p)	6.92	7.47
H661	LC	wt	wt	wt	wt	wt	t(3p13;5p15)	8.43	8.16

### Induction of apoptosis by TUSC2-MK2206 in TUSC2/LKB1-defective NSCLC cell lines involves caspase-9 activation

**Figure 3:**
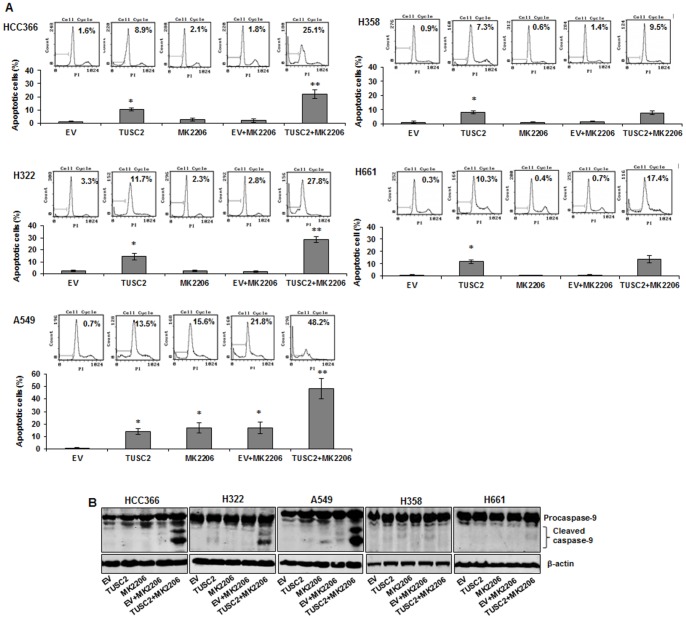
Apoptosis mediated by TUSC2 transfection and MK2206 combined treatment involves caspase-9 activation. Cells were transfected with DC-TUSC2 for 24 hours, starved for 24 hours and then treated with 1 µM MK2206 for 48 hours before measuring their apoptotic activity. A) DNA fragmentation was analyzed by flow cytometry, and relative apoptotic cells were calculated in terms of the FITC-positive values in cells. Columns, mean of three different experiments, each with duplicate samples; bars, SD. *, *P*<0.05, compared with EV control; **, *P*<0.05, compared with EV+MK2206; B) Proteins in cell lysates were separated with 12% SDS PAGE, probed with caspase-9 antibody to detect both full length caspase-9 (47 kDa) and large fragments of caspase-9 (35 kDa, 37 kDa). Immunoreactive bands were visualized with an Odyssey Imager.

To further evaluate the biological activity of TUSC2-MK2206 combined treatment in the three tested LKB1-defective NSCLC cell lines, we measured induction of apoptosis using cell cycle analysis with fluorescence-activated cell sorting (FACS). We found that TUSC2 transient expression, alone, caused a 10-15% increase of apoptosis in all three cell lines. MK2206 treatment alone enhanced apoptosis in A549 by 17%, but had no significant effect on HCC366 or H322. However, when cells were transfected with 2 µg TUSC2 and treated with 1 µM MK2206, apoptosis was significantly increased (*P*<0.05). The relative apoptotic rates in HCC366, H322, and A549 cells increased by 25%, 37%, and 50%, respectively (Fig. 3A). In contrast TUSC2-MK2206 combined treatment had no significant effect on LKB1-wild type H358 and H661 cells (Fig.3A).

Activation of caspase-9 is a key factor in the mitochondrial apoptotic pathway driven by tumor suppressors. To determine whether caspase-9 was involved in the observed increase of apoptosis by TUSC2-MK2206, we analyzed its cleavage by Western blot. Figure 3B shows that when TUSC2/LKB1-defective HCC366, H322, and A549 cells were treated with either TUSC2 or MK2206, alone, cleavage of caspase-9 was either very low or no detectable. In contrast, when the cells were exposed to the combination of TUSC2 and MK2206 treatment, caspase-9 was robustly cleaved from its inactive precursor form (procaspase-9, 47 kDa) into active fragments (cleaved caspase-9, 35/37 kDa). TUSC2-MK2206 combined treatment did not result in detectable levels of caspase-9 cleavage in TUSC2-defective/LKB1-wild type H358 and H661 cells (Fig. 3B).

### In vivo tumor growth inhibition by TUSC2-MK2206 in a human TUSC2/LKB1-defective H322 lung cancer xenograft mouse model

**Figure 4:**
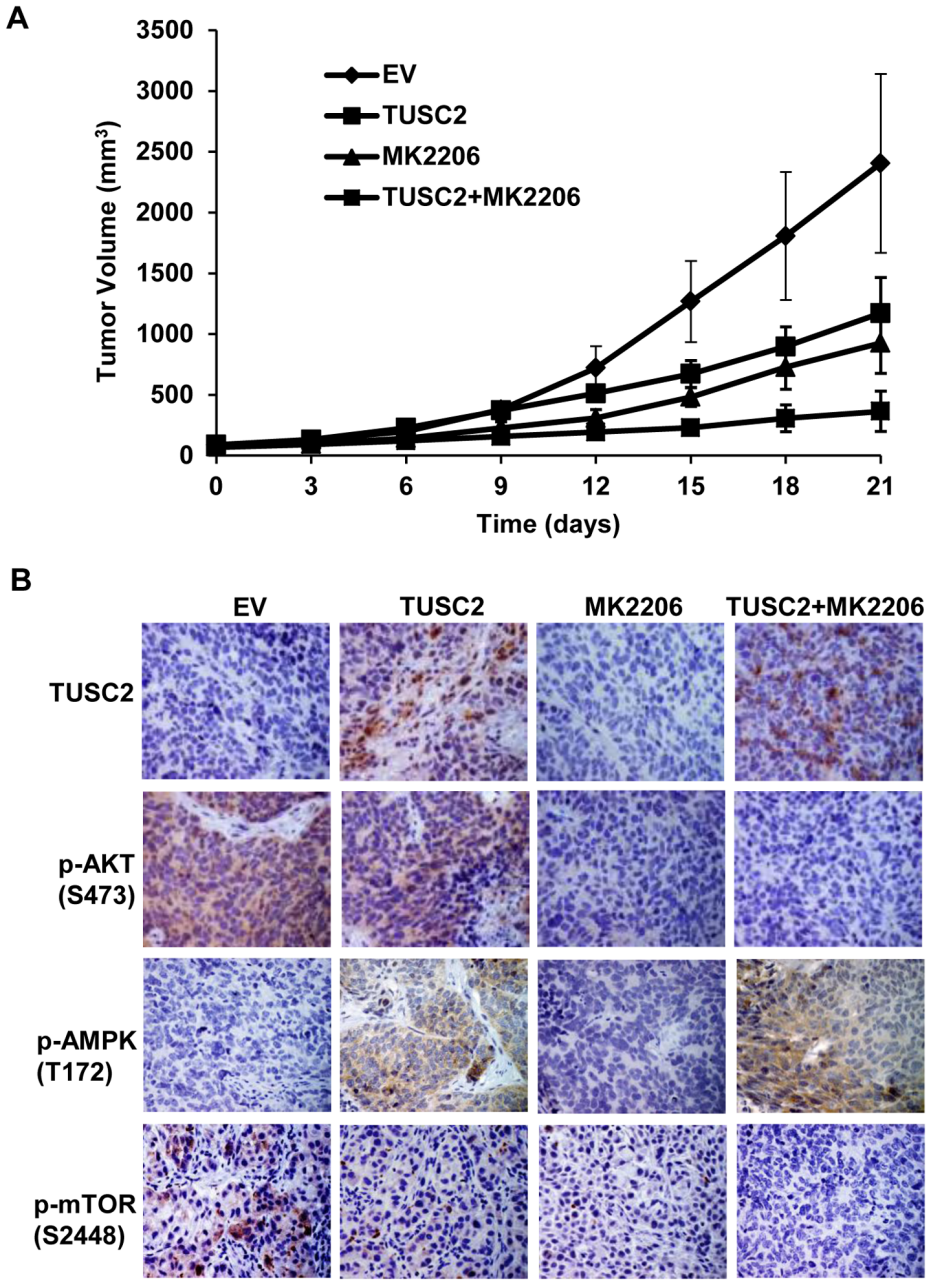
Effective *in vivo* inhibition of tumor growth by TUSC2 systematic restoration and MK2206 combined treatment. A subcutaneous mouse model of human NSCLC H322 was used to evaluate the combined effect of systemic delivery of the DC–based TUSC2 nanoparticles and MK2206 treatment on tumor growth inhibition. A) Tumor volume was calculated, taking length to be the longest diameter across the tumor and width to be the corresponding perpendicular diameter, using the following formula: length × width^2^×0.52. Tumor growth inhibition rate was calculated as 100%× (tumor size_treated_/tumor size_control_) on each measurement day. Bars, SD; B) Tumors were resected, fixed with 4% paraformaldehyde, paraffin-embedded for immunohistochemistry staining with the indicated antibodies, and examined under a Nikon TC200 fluorescence microscope equipped with a digital camera.

Previously, we have demonstrated the efficacy of intravenous systematic nanovesicle TUSC2 gene delivery in NSCLC mouse models [4]-[7]. To validate the antitumor activity of TUSC2-MK2206 *in vivo*, we evaluated the effect of this combination on inhibiting tumor growth in an LKB1-defective human H322 lung cancer xenograft mouse model. Mice with established flank tumors of equal volumes were divided into different treatment groups: DC-EV complex; DC-TUSC2 complex; MK2206 alone; and DC-TUSC2 complex plus MK2206. The overall effects of treatments on tumor growth were analyzed by measuring tumor size at three day intervals. Figure 4A shows that the combination of TUSC2 intravenous injection and MK2206 treatment was significantly superior (*P*<0.05) in reducing tumor volumes than either agent alone. The mean tumor volume was 363.8±165.4 mm^3^, compared with 1171.5±293.7 mm^3^ and 926.4±250.5 mm^3^ in those groups receiving intravenous TUSC2 or MK2206 treatment alone, respectively. Immunohistochemical (IHC) staining of tumor tissue sections showed effective TUSC2 delivery to the lungs and marked inhibition of p-AKT expression by MK2206 (Fig. 4B). The combination therapy group induced no apparent weight loss and change in food and water intake compare to control groups.

### Stimulation of AMPK phosphorylation and kinase activity by TUSC2

**Figure 5:**
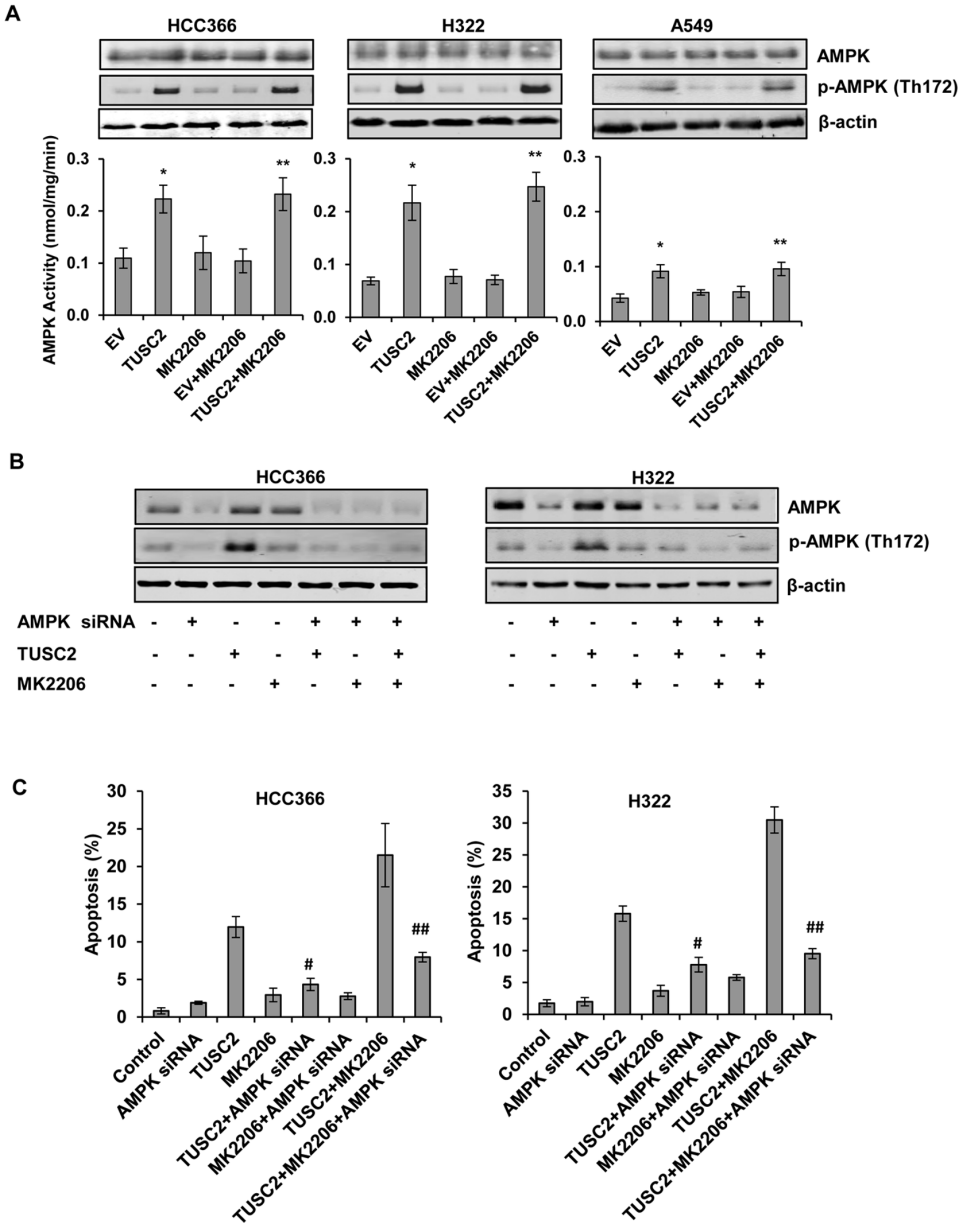
Stimulation of AMPK phosphorylation and kinase activity by TUSC2 in LKB1-defective cells. A) HCC366, H322, and A549 were transfected with TUSC2 then either treated with 1 µM MK2206 for 24 hours or left untreated. AMPK kinase activity in the immunocomplexes was measured by phosphorylation of SAMS peptide as described in Materials and Methods. Columns, mean of three different experiments, each with duplicate samples; bars, SD. *, *P*<0.05, compared with EV control; **, *P*<0.05, compared with EV+MK2206. LKB1- defective cells HCC366 and H322 cells were co-transfected with 2 µg TUSC2 plasmid and 50 nM AMPK siRNA with Lipofectamine™ 2000. Twenty-four hours after transfection, cells were starved for 24 hours and treated with 1 µM MK2206 for an additional 48 hours. B) Cell lysis were collected for western blot analysis to assess levels of AMPK and p-AMPK proteins; or C) Cells were assayed for apoptosis as described in Materials and Methods. Columns, mean of three different experiments, each with duplicate samples; bars, SD. ^#^, *P*<0.05, compared with TUSC2; ^##^, *P*<0.05, compared with TUSC2+MK2206.

To test whether TUSC2-MK2206 combined treatment of LKB1-defective cells HCC366, H322, and A549 influenced AMPK activation, we analyzed AMPK phosphorylation levels and kinase activity by immunoblot and *in vitro* kinase assays. Untransfected cells and cells transfected with either TUSC2 or treated with MK2206 alone served as controls. We found that TUSC2, alone, significantly increased AMPK phosphorylation and kinase activity (*P*<0.05) (Fig. 5A). In contrast, MK2206, alone, had no noticeable effect on this activity. Stimulation of AMPK phosphorylation and kinase activity by TUSC2-MK2206 in all three cell lines was slightly higher than that of TUSC2 alone.

In vivo, expression levels of phospho-AMPK proteins, in the excised xenograft tumor tissues, were assessed by immunohistochemical (IHC) staining using p-AMPK (T172) antibody. Similar to the *in vitro* results, TUSC2 systemic delivery, alone, led to pronounced levels of APMK phosphorylation. MK2206 had no significant effect on this activity. P-AMPK levels in tumors treated with TUSC2-MK2206 were slightly higher than those of TUSC2 alone (Fig. 4B).

### TUSC2-MK2206 cooperative tumor cell growth inhibition requires AMPK expression

To further understand the role of AMPK in TUSC2-enhanced sensitivity to MK2206, we suppressed AMPK expression by gene knockdown, and assessed growth and survival of LKB1-defective HCC366 and H322 cells after TUSC2-MK2206 treatment. We identified an AMPK siRNA that inhibited AMPK expression and phosphorylation (Figure 5B). AMPK siRNA co-transfection of HCC366 and H322 cells reduced TUSC2-induced apoptotic activity by 50%. More importantly, AMPK knockdown interfered with TUSC2-MK2206 cooperative induction of apoptosis, suggesting that in these cells, AMPK expression contributed to the observed enhanced MK2206 sensitivity by TUSC2.

### TUSC2-MK2206 inhibition of AKT phosphorylation and kinase activity

**Figure 6:**
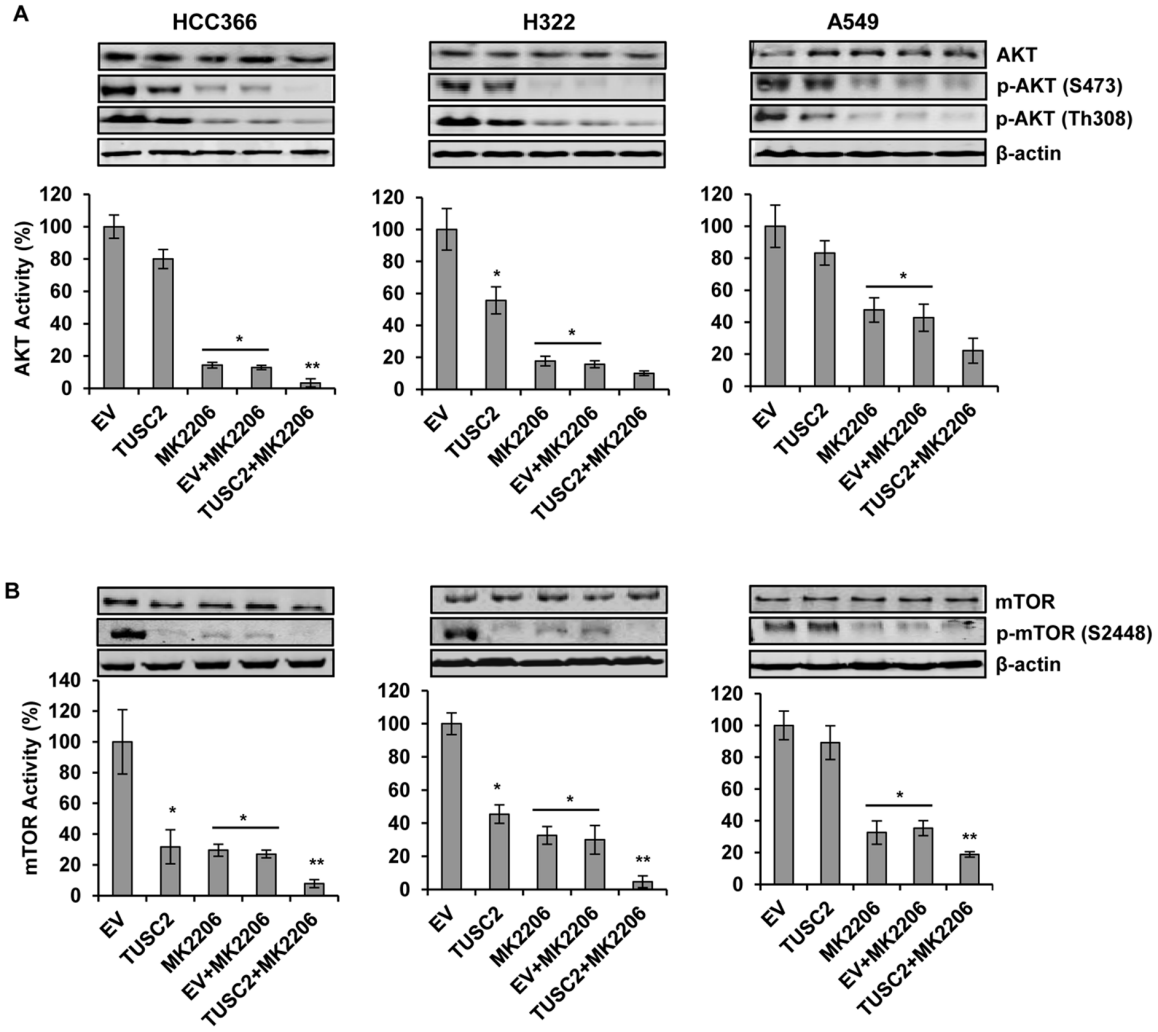
Inhibition of AKT and mTOR kinase activity by TUSC2 transfection and MK2206 combined treatment. LKB1-defective HCC366, H322, and A549 were transfected with TUSC2 for 24 hours, starved for 24 hours and either treated with 1 µM MK2206 for 24 hours or left untreated. Cell lysates were collected for western blot analysis for the levels of A) p-AKT(S473) and p-AKT(Th308); and B) p-mTOR(S2448). AKT and mTOR was precipitated from 200 ug cell lysis using A) AKT or B) mTOR antibodies. The kinase activity of AKT and mTOR were measured with KLISA AKT and mTOR assay kit, respectively, using GSK-3α and S6K GST fusion proteins as substrates, respectively. Kinase activities were determined by ELISA, as substrate absorbance was measured at 450 nm, and reference wavelengths were measured at 540/595 nm using a Synergy 2 Multi-detection microplate reader. Columns, mean of three different experiments, each with duplicate samples; bars, SD. *, *P*<0.05, compared with EV control; **, *P*<0.05, compared with EV+MK2206.

Since MK2206 is a selective inhibitor of AKT signaling, we analyzed whether the transfection of TUSC2 would further inhibit AKT phosphorylation and kinase activity *in vitro* and *in vivo*. Using two different p-AKT antibodies, S473 and Th308, we found that MK2206 treatment, alone, effectively inhibited AKT phosphorylation levels in all three LKB1-defective cell lines. The AKT kinase activity, in HCC366, H322, and A549 cells, was inhibited by 86%, 82%, and 52%, respectively (Fig. 6A). TUSC2, alone, reduced this activity by 20%, 44%, and 17%, respectively. However, the combination of TUSC2 transfection and MK2206 treatment inhibited AKT kinase activity by 97%, 90%, and 78%, respectively, which was higher than either agent alone.

In vivo, AKT phosphorylation, in the excised xenograft tumor tissues, was assessed by immunohistochemical (IHC) staining using p-AKT (S473) antibody. Similar to the *in vitro* results, while TUSC2 systemic delivery alone led to a slight decrease of AKT phosphorylation, MK2206 significantly inhibited this activity (Fig. 4B). P-AKT levels in tumors treated with TUSC2-MK2206 were lower than those of MK2206 alone.

### TUSC2-MK2206 inhibition of mTOR phosphorylation and kinase activity

Signaling through AKT can be propagated to a diverse array of substrates, including the downstream effector mTOR, a key regulator of protein translation [25]. AMPK mediates mTOR activation via AKT [28], which regulates tumor cell growth through the inhibition of the mammalian target of rapamycin (mTOR) pathway. Therefore, we assessed the effect of the combination of TUSC2 transfection and MK2206 treatment on mTOR phosphorylation and kinase activity *in vitro* and *in vivo*. In vitro, TUSC2 or MK2206 treatment alone on LKB1-defective HCC366, H322, and A549 cells significantly reduced the phosphorylation levels of mTOR (Fig. 6B). The kinase activity of m-TOR was inhibited by 70%, 55%, and 11% after TUSC2 transfection alone; and by 72%, 65%, and 65% when treated with MK2206 alone. The combination treatment reduced mTOR activity by 92%, 95%, and 81%, respectively.

In vivo, mTOR phosphorylation, in the excised xenograft tumor tissues, was assessed by immunohistochemical (IHC) staining using p-mTOR (S2448) antibody. Similar to the *in vitro* results, TUSC2 systemic delivery or MK2206 treatment alone led to a significant decrease of mTOR phosphorylation (Fig. 4B). P-mTOR levels in tumors treated with TUSC2-MK2206 were lower than those of either agent alone. Taken together, these results demonstrate a cooperative inhibition of mTOR enzymatic activity by TUSC2-MK2206 combined treatment.

## Discussion

Drug resistance is a major cause of treatment failure for patients with lung cancer. We have recently investigated the possibility that transfect of the tumor suppressor gene TUSC2 in NSCLC cells deficient of its expression, could potentiate sensitivity to small-molecule targeted cancer therapy. Based on the notion that TUSC2 gene therapy combined with specific pro-apoptotic stimuli may have a therapeutic value, we assessed TUSC2 functional effects on AKT inhibition by MK2206. Targeting this critical converging signaling node is relevant because the AKT pathway is an important regulator of cell growth and survival. AKT is frequently deregulated in human cancers and its constitutive signaling has been found to correlate with reduced sensitivity to antitumor drugs [29]. To rule out the possibility that the effect of TUSC2 and MK2206 combined treatment could be a cell line or a gene-specific effect, we used multiple TUSC2-deficient NSCLC cell lines from various cell types with varying genetic background (Table 1). In addition, we used a xenograft mouse model of the human lung cancer cell line H322, which is LKB1-defective, to determine whether TUSC2 systemic nanovesicle-based gene delivery and MK2206 treatment would effectively inhibit xenograft tumor growth. Mice tolerated the combined treatment very well without any apparent toxicity or adverse effects.

Although dose-response curves showed that the optimal response to MK2206 was observed at 5 µM without any cell toxicity, experiments were conducted with only 1 µM MK2206 to demonstrate that TUSC2-MK2206 combined treatment induced functional biological responses at low concentrations achievable in patients. In this report, we showed that the combination of TUSC2 transfection and MK2206 treatment suppressed tumor cell viability *in vitro* and effectively inhibited xenograft tumor growth *in vivo* more effectively than either agent alone. TUSC2 and MK2206 induced tumor cell apoptosis via activation of caspase-9, as shown by its cleavage, however, whether this caspase is an essential downstream component of this activity is yet to be determined.

An intriguing finding was that enhanced sensitivity to MK2206 by TUSC2 was influenced by the expression status of the tumor suppressor gene LKB1. The molecular linkage between LKB1 inactivation and TUSC2-MK2206 cooperation remains to be determined. LKB1 is inactivated in up to 50% of NSCLC, and TUSC2 expression is either reduced or completely lost in 82% of NSCLC and 100% of SCLC [2], [3], [27]. In this regard, one plausible explanation for the observed LKB1 connection to TUSC2-enhanced sensitivity to MK2206 is that LKB1 may be an integral component of a broad phenotype of interconnecting TUSC2-dependent apoptotic pathways. This is similar to other studies that have reported regulation of p53-dependent apoptosis by LKB1 [30], and in accord with our previous findings that TUSC2 and p53 cooperatively induced apoptosis in both p53-sensitive and p53-resistant NSCLC cell lines [8].

Since the primary action of LKB1 function is through direct activation of AMPK, thereby regulating apoptosis in response to energy stress [26], [27], we investigated the involvement of AMPK in the interaction between TUSC2 and MK2206. Whereas, MK2206 alone had no effect on AMPK phosphorylation or kinase activity, TUSC2 transfection alone profoundly stimulated this activity. More importantly, when we suppressed AMPK expression, through gene knock down, TUSC2-MK2206 induction of apoptosis was greatly reduced. These results are mechanistically informative and relevant for several reasons. First, to our knowledge, this is the first study showing AMPK activation by TUSC2. Second, the direct correlation between AMPK expression and MK2206-TUSC2 cooperation illustrates a regulatory role for AMPK in enhanced sensitivity to MK2206 by TUSC2. Third, the efficacy of the combination of TUSC2 transfection and MK2206 treatment required inactive LKB1, not its wild-type genomic counterpart. Taken together, these functional findings suggest that TUSC2 might function in a similar fashion as LKB1 in regard to AMPK activation. The fact that TUSC2 transfection stimulated AMPK phosphorylation at Th172, the same residue used by LKB1 proteins for coupling with AMPK, raises the possibility that TUSC2 and LKB1 might compete with each other for binding to the same functional protein-binding site on AMPK. Thus, these interactions would change the dynamic of AMPK signaling with regard to MK2206 sensitization [26]. In this case, when LKB1 is inactivated, such as in H322, HCC366, and A549, LKB1-AMPK coupling did not occur. Instead, unchallenged TUSC2, directly or indirectly, activated AMPK at the same sites that would normally be targeted by LKB1. This dynamic would explain why TUSC2 transfection was effective in enhancing sensitivity to MK2206 in LKB1-defective cells, but not in the LKB1-wild type H358 and H661 cells.

Next, in an attempt to identify the regulatory networks of TUSC2 signaling activity, we analyzed the correlation between TUSC2-MK2206 activity and the activation status of the AKT/mTOR axis in LKB1-defective cells. LKB1 plays an opposite role to AKT by negatively regulating mTOR through the activation of AMPK [26], [28]. We found that TUSC2 transfection alone resulted in a moderate inhibition of AKT phosphorylation and kinase activity in all three cell lines. Furthermore, TUSC2 markedly inhibited the phosphorylation levels of mTOR, as well as its kinase activity in HCC366 and H322, but just slightly in A549. As expected, mTOR was significantly inactivated by MK2206 alone. TUSC2 and MK2206 combined treatment suppressed the kinase activity of AKT and mTOR, respectively to a greater extent than each single agent. These results indicated that MK2206 inhibition of AKT and mTOR activation could be further enhanced by TUSC2 transfection, confirming the strategy of dual downstream target inhibition converging on the AKT pathway.

Taken together, the central and novel finding in the present study demonstrates the efficacy of TUSC2 transfection in enhancing tumor cell sensitivity to MK2206 treatment. AMPK activation and inhibition of AKT and mTOR kinases appear to be involved in regulating the observed LKB1-dependent TUSC2-MK2206 synergy, although, it is possible that other signaling modules might also be involved. This study provides the molecular rationale and biomarker profile for combining TUSC2 gene therapy with kinase inhibitors for more effective treatment of lung cancer.
